# Long-term survival from multidisciplinary treatment of primary malignant pericardial mesothelioma: A case report

**DOI:** 10.1016/j.ijscr.2024.110615

**Published:** 2024-11-15

**Authors:** Vu Anh Hai, Nghiem Thi Minh Chau, Ho Viet Hoanh, Ha Van Tri, Dinh Cong Pho, Nguyen Van Nam

**Affiliations:** a Oncology Center, Military Hospital 103, Vietnam Military Medical University, Hanoi 10000, Viet Nam; b Department of Cardiology, Heart Institute, 108 Military Central Hospital, Hanoi 100000, Viet Nam; c Department of Thoracic Surgery, Military Hospital 103, Vietnam Military Medical University, Hanoi 10000, Viet Nam

**Keywords:** Primary malignant pericardial mesothelioma, Long-term survival, Multidisciplinary treatment

## Abstract

**Introduction and importance:**

Primary malignant pericardial mesothelioma is an extremely rare disease with a poor prognosis. Currently, there are no specific guidelines for diagnosis and treatment in Vietnam and international countries for this disease, so treatment experiences from previous clinical cases are very important in the management of this disease.

**Case presentation:**

The research team reports a clinical case diagnosed and treated with a combination of surgery and chemotherapy using the Pemetrexed + Carboplatin regimen at the Oncology Center. The patient has survived for an additional 17 months up to the current time.

**Clinical discussion:**

This is an exceptionally rare incident. He survived for a duration of 17 months, which is almost three times longer than the average survival time observed in other documented cases. Additional reports on cases with pericardial mesothelioma are necessary to have a comprehensive understanding of its nature, enabling early identification and appropriate treatment.

**Conclusion:**

The patient underwent surgical intervention and received chemotherapy as part of their treatment. The research team emphasizes the importance of a multidisciplinary approach in diagnosing and treating primary malignant pericardial mesothelioma.

## Highlights

• Primary malignant pericardial mesothelioma is a extremely rare disease with a poor prognosis and no specific guidelines.
• The case was treated with a combination of surgery and chemotherapy. The patient has survived for along time.
• The research team emphasizes the importance of a multidisciplinary approach in the diagnosis and treatment of this disease.

## 1 Background

Primary malignant pericardial mesothelioma is an exceptionally uncommon form, comprising approximately 0.7 % of all mesothelioma cases [1]. This cancer has a poor prognosis, with a median survival period of roughly six months from the time of diagnosis [2]. Currently, there is no definitive diagnostic and treatment protocol for this disease, both globally and in Vietnam. Diagnosis and therapy primarily rely on clinical instances documented in the medical literature. So far, the number of reported cases worldwide is approximately 200 [3]. We have recently acquired a case of pericardial mesothelioma at the Oncology Centre. To enhance our expertise in detecting and treating this ailment, we documented the case and examined the medical literature. This manuscript was prepared in accordance with the SCARE 2023 guidelines [4].

## 2 Case presentation

A 50-year-old male patient with no known prior health issues and no documented exposure to asbestos was admitted to the hospital on May 18, 2022, for emergency treatment. The patient presented with symptoms including exhaustion, shortness of breath, chest pain, and other related symptoms. Before hospitalisation, significant advancements were occurring approximately one week earlier. The patient presented with no prior history of chronic illness or cardiovascular disease, nor did they report any unusual symptoms in the week leading up to hospital admission. During this pre-admission period, the patient maintained normal physical activities and routine exertion levels without signs of impairment. However, the clinical course of the illness advanced rapidly over the first week, culminating in an emergent hospital admission necessitated by acute symptoms indicative of cardiac tamponade. Upon admission, the patient exhibited classic features of cardiac tamponade alongside accompanying symptoms of mild fever and dyspnea*.* Initial assessment upon admission: The patient presents with a fever of 37.8 degrees Celsius, a respiratory rate of 24 breaths per minute, well-ventilated lungs, oxygen saturation of 98 %, dilated jugular veins while lying down, tachycardia of 110 beats per minute, and a blood pressure of 95/60 mm Hg.

Medical tests revealed an enlarged heart silhouette and two well-illuminated lung regions on the chest X-ray. Echocardiography indicated significant pericardial effusion, resulting in the right ventricle and right atrium compression. No damage was detected during the abdominal ultrasound examination. The patient had emergency pericardial draining, during which 1000 ml of blood serum was extracted. The results of the AFP test and pericardial fluid culture were both negative. The cytological analysis of the pericardial fluid reveals the presence of chronic inflammatory fluid. The sample of pericardial fluid displays clusters of abnormal epithelial cells with heightened pigmentation and coarse nuclear material. Immunohistochemistry results indicate diffuse and high positivity for CK5/6, CK7, and Calretinin and moderate and diffuse positivity for WT1. However, the results are negative for TTF-1. These findings suggest the presence of pericardial mesothelioma.

**Fig. 1:**
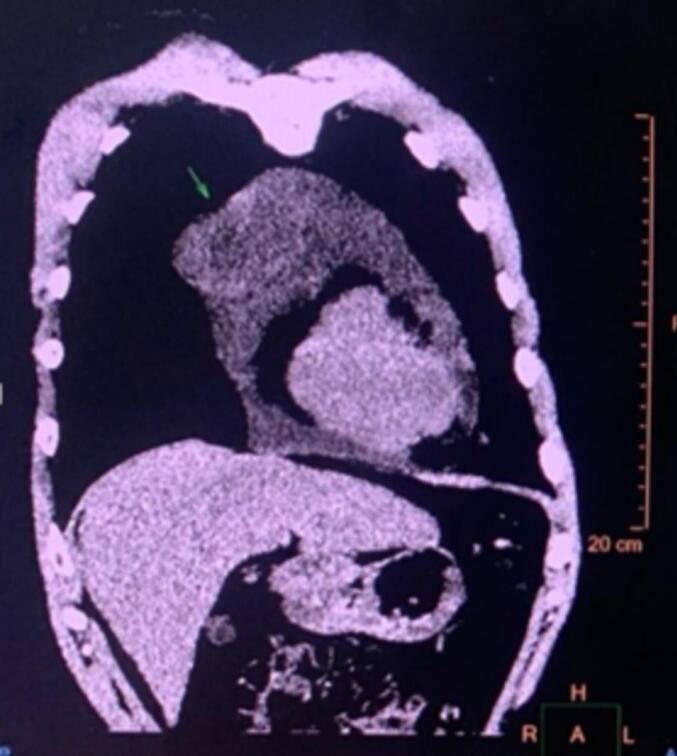
Image obtained before surgery showing a tumor in the pericardium.

To thoroughly assess the lesion and differentiate it from metastatic cancer in the pericardium, the patient underwent a chest CT and PET/CT scan. The findings revealed the presence of a tumor in the pericardium, thickening of the pericardium in a nodular pattern, and increased uptake of FDG. Additionally, there was a moderate amount of fluid accumulation in the pericardium. No evidence of damage to other organs was observed (Fig. 1).

The patient underwent a surgical procedure to perform a biopsy and partially remove a tumor located in the pericardium. Additionally, a pericardial-pleural window was created. Preoperative imaging assessments, including CT and PET/CT scans, indicated the clinical necessity for a definitive diagnosis, thus necessitating surgical intervention for this patient. The surgical approach sought to achieve three main objectives. Initially, a surgical biopsy is performed to obtain tissue for histopathological analysis, facilitating an accurate diagnosis that will guide future treatment planning. Secondly, the creation of a pericardial window is performed to relieve cardiac tamponade and support continued therapeutic management. Thirdly, lesions are resected based on intraoperative evaluation of gross and histological findings. This comprehensive surgical approach addressed the urgent cardiac tamponade while also enhancing diagnostic clarity and establishing optimal conditions for subsequent treatment.

**Fig. 2:**
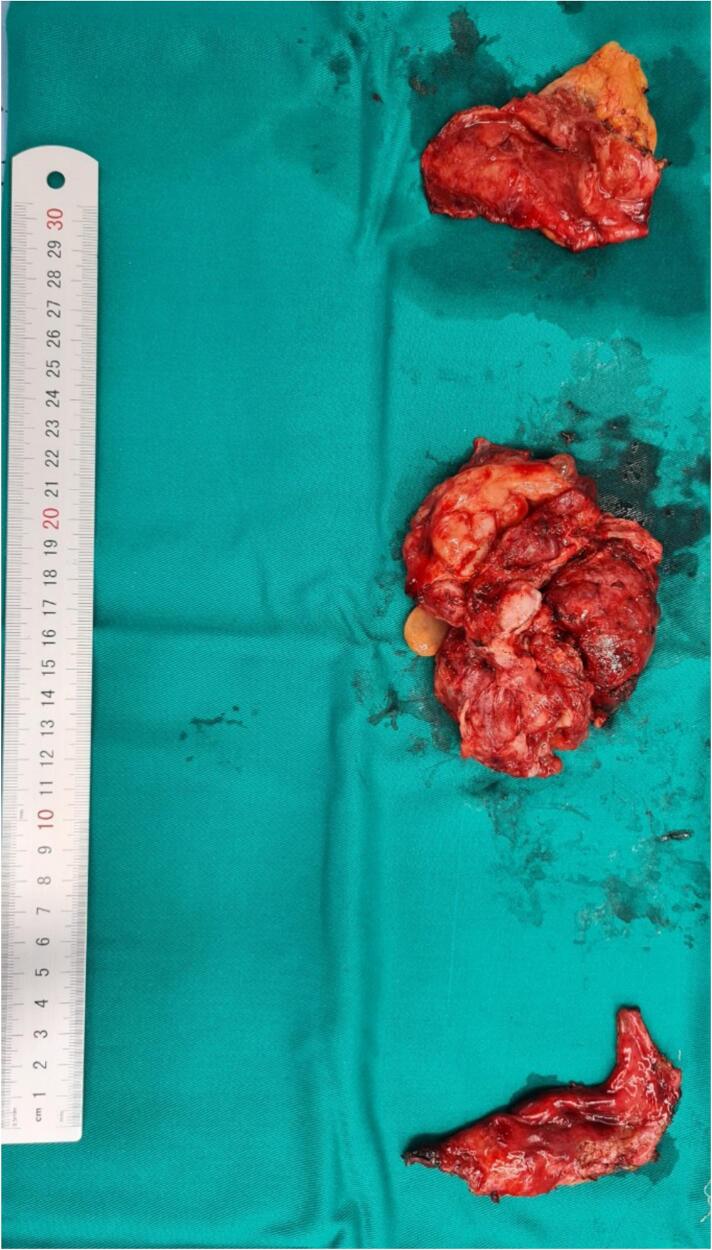
Image of the resected pericardial tumor.

During surgery, after specimen collection, an immediate histopathological examination was conducted via frozen section biopsy, confirming the diagnosis intraoperatively. Maximal resection of the pathological tissue was pursued wherever possible. The tumor's invasive characteristics, which involve encasing and infiltrating essential cardiac structures, made complete excision highly difficult. A pericardial window was established into the left pleural cavity, and partial tumor reducing was performed within safe margins to protect critical structures. This method emphasized surgical safety while focusing on the main objectives of relieving cardiac compression and enhancing post-operative care. Numerous small nodular infiltrates were also present. Additionally, a massive tumor measuring 15 × 15 cm was found on the front of the heart. This tumor had invaded the surrounding tissues and was causing compression of the superior vena cava (Fig. 2).

Following the surgical procedure, the patient's condition remained stable. They no longer had shortness of breath or chest tightness. The neck veins were no longer distended, and the heart rate ranged between 80 and 86 beats per minute. The blood pressure was recorded as 110–120/80 millimeters of mercury.

**Fig. 3:**
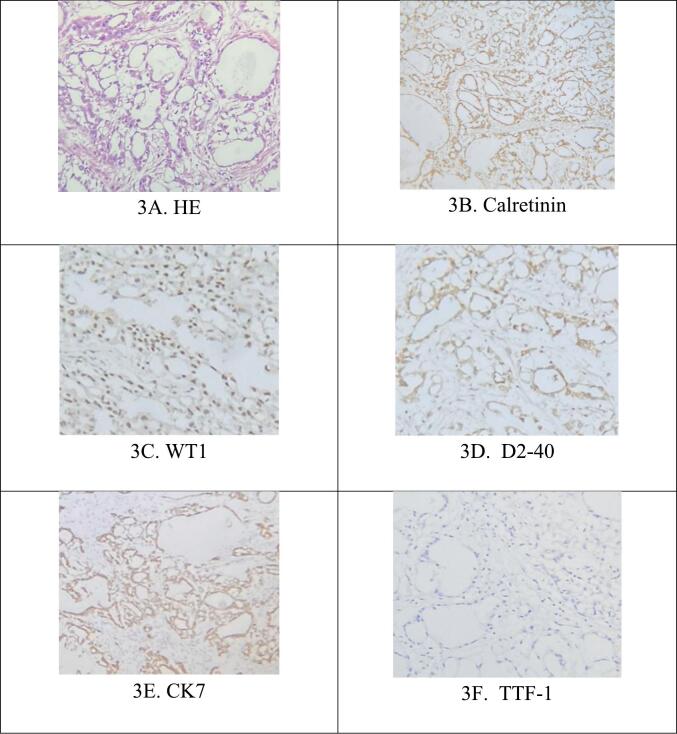
Pathological results.

The pathological findings post-surgery reveal that the tumor is composed of rapidly dividing cells that form both big and small ductal structures. Some areas of the tumor contain mucus, irregular cell nuclei, enhanced pigmentation, and minimal cytoplasm. Additionally, the tumor has invaded the fibrous connective tissue surrounding the pericardium. Immunohistochemistry results showed a widespread and intense presence of CK7, D2-40, and calretinin. There was also a moderate and widespread presence of WT1. However, TTF-1, CDX-2, and CK 20 were not detected. Diagnosis confirmed: Epithelioid pericardial malignant mesothelioma. The patient was administered a chemotherapy regimen consisting of Pemetrexed at a dose of 500 mg/m2 and Carboplatin at a dose of AUC 6, with a cycle duration of 21 days (Fig. 3).

**Fig. 4:**
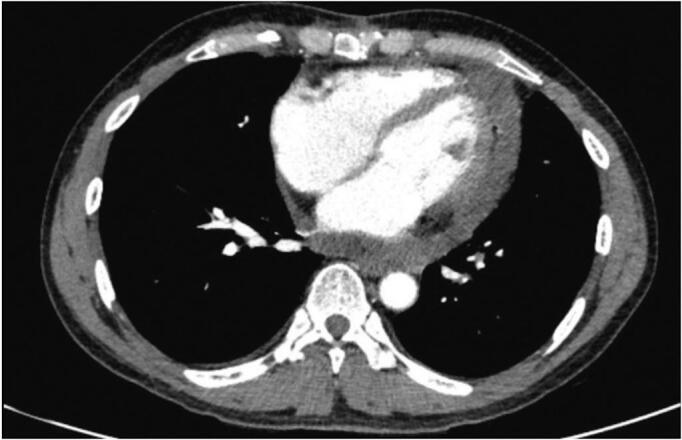
Chest CT (October 2022) with mild pericardial effusion.

Process of therapy and treatment efficacy: Following six cycles of chemotherapy, the patient's clinical state is stable. They do not experience any trouble breathing or chest pain. Additionally, there is no collapse of the neck veins when lying down. The patient's pulse rate ranges from 80 to 88 cycles per minute, and their blood pressure ranges from 120 to 130 mm Hg systolic and 70 to 80 mm Hg diastolic (Fig. 4).

**Fig. 5:**
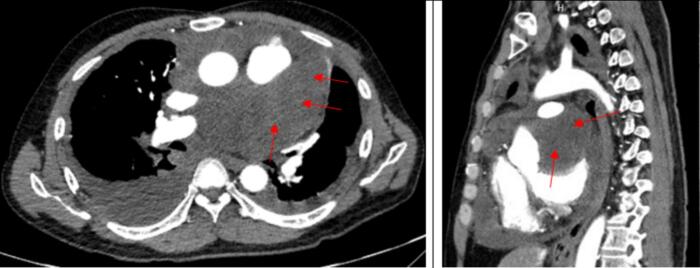
Chest CT (September 2023): bilateral pleural effusion, large tumor in the base area around large blood vessels, pericardial effusion.

Nevertheless, as a result of grade 2–3 leukopenia, the patient was transferred to continue receiving Pemetrexed monotherapy for an additional four cycles. By January 2023, the disease had progressed to pleural metastasis and bilateral pleural effusion, and there was an increase in the size of tumors in the pericardium, located at the base of the heart. The treatment was modified to include the chemical regimen Gemcitabine at a dosage of 1000 mg/m2 on day 1, for 8 cycles lasting 21 days each, repeated 3 times. However, the disease did not show any response to this treatment, and as a result, the patient was moved to receive comprehensive palliative care treatment. The patient, who has been diagnosed with cancer for 17 months, is currently experiencing advanced symptoms of the disease, including physical wasting, difficulty breathing, chest pain, significant pleural effusion on both sides (requiring regular pleural puncture), moderate pericardial effusion, tachycardia (heart rate of 100–120 bpm), and multiple large tumors near the base of the heart and surrounding major blood vessels. Treatment is ongoing to manage the symptoms (Fig. 5).

## 3 Discussion

The successful outcome in this case can be attributed to a combination of key factors: accurate diagnosis, effective intervention, and careful post-operative management. The initial precise diagnosis enabled targeted treatment of cardiac tamponade, which was successfully managed through a surgical pericardial window procedure. Following surgery, the patient remained stable, with no recurrence of cardiac compression or associated complications. Additionally, left pleural effusion, likely secondary to the drainage of pericardial fluid, was minimal and manageable, allowing for uncomplicated clinical monitoring and intervention as needed. The adjunctive use of chemotherapy was instrumental in controlling the underlying disease process, slowing its progression, and stabilizing the patient's overall condition. As a result, the patient's general health improved, permitting adherence to and tolerance of the prescribed treatment regimen. These combined therapeutic efforts underscore the importance of a comprehensive and interdisciplinary approach, which ultimately contributed to an improved prognosis and quality of life for the patient.

Pericardial mesothelioma is a malignancy that originates from the cells of the pericardial mesothelium. This malignancy is an exceptionally uncommon, representing just 0.7 % of all mesothelioma occurrences [1]. Up to this point, the number of recorded cases worldwide is approximately 200. [3] Consequently, just a small number of physicians come across patients suffering from pericardial mesothelioma in the course of their professional duties. Prevalence of the condition is higher in males than females, with the typical start age occurring at 46 years [2,5].

Pericardial mesothelioma frequently advances without noticeable symptoms, making it challenging to detect in its early stages. Patients with constrictive pericarditis, pericardial effusion, cardiac tamponade, invasive tumors causing compression of blood vessels and surrounding tissues, and heart failure are frequently admitted to the hospital and diagnosed at an advanced stage. They commonly experience functional symptoms such as fatigue, shortness of breath, chest pain, and palpitations [3,6,7]. Pericardial mesothelioma is classified into three kinds based on the location and extent of tumor spread: localized, diffuse with many nodular lesions in the pericardium surrounding the heart, and the ability to disseminate, invade, and metastasis to organs outside the heart [8]. Pericardial mesothelioma can be classified into three forms at a microscopic level: epithelial type, connective tissue type, and mixed epithelial-connective tissue type. Among these, the epithelial type is the most prevalent and also has a favourable prognosis [9,10].

The prognosis for pericardial mesothelioma is extremely poor because the majority of cases are diagnosed in advanced stages, rendering significant surgery impossible. Additionally, the illness exhibits minimal response to chemotherapy, radiation, and other treatment options. Using the treatments currently accessible, the average length of time a person survives from the moment of diagnosis is approximately six months. Patients frequently succumb to constrictive pericarditis, cardiac tamponade, heart failure, and arrhythmia [2,11].

Extensive evidence has shown that asbestos as the primary and most significant risk factor for pleural and peritoneal mesothelioma. Nevertheless, the connection between Asbestos and pericardial mesothelioma remains disputed and lacks definitive evidence, primarily due to the limited number of clinical cases identified compared to mesothelioma affecting pleural and peritoneal tissues. Several studies indicate that the incidence of pericardial mesothelioma associated with asbestos exposure is relatively low [5]; concurrently, several other investigations have discovered that the vast majority of pericardial mesothelioma cases are associated with asbestos exposure [12]. Additional potential risk factors include: exposure to radiation, infection with tuberculosis, and presence of the SV40 virus [11]. Here, the patient has a healthy medical history, is employed as a driver, and has not been in contact with asbestos, such as through water from fibro-cement roofs or fire-resistant substances. The patient had no prior TB or exposure to ionizing radiation. This is likely a case of pericardial mesothelioma where no identifiable risk factors have been found.

Like several documented instances, this patient received a diagnosis during an advanced stage and was taken to the hospital while experiencing evident symptoms, including chest tightness and difficulty breathing, in the setting of pericardial effusion. At high levels, it induces cardiac tamponade. Initial diagnostic examinations, such as chest X-ray and abdomen ultrasound, did not reveal any organ damage other than the already identified ones. Given the uncommon nature of pericardial mesothelioma, it is essential first to consider other more prevalent causes, such as acute pericarditis or pericardial TB, when making a diagnosis. Pericardial metastasis refers to the spread of malignancy from its original site, particularly to the pericardium. Pericardiocentesis is performed to alleviate cardiac tamponade and provide fluid for diagnostic testing such as bacterial culture, tuberculosis screening, cytology, and cell-block analysis. Based on statistics, the incidence of pericardial mesothelioma patients with malignant cells discovered in their drainage fluid is relatively low, at approximately 20 % [5].

Consequently, numerous instances have occurred when patients were initially misdiagnosed and treated for pericardial TB, only to receive a clear diagnosis of pericardial mesothelioma later. This case might be described as one where the patient had a relatively positive diagnostic process. The Cell-Block test revealed the presence of cancerous cells in the pericardial fluid, and the immunohistochemistry results were likewise typical, showing the presence of cancerous cells. The specimen exhibits a pronounced and widespread presence of CK5/6, CK7, and Calretinin and a moderate and diffuse presence of WT1. However, it does not show any presence of TTF-1. The presence of malignant cells in pericardial fluid is likely associated with the pericardium's quantity and extent of cancerous formations. The chest CT, PET/CT, and surgeon's notes revealed that the pericardial tumor was significantly big and had widespread nodular infiltrates in the pericardium, indicating a late-stage diagnosis.

There is no established and universally accepted treatment protocol for pericardial mesothelioma. Therefore, the approach to treating these cases mostly relies on the regimen used for pleural and peritoneal mesothelioma and the knowledge gained from previous experiences. Individualised clinical cases have been documented, customised to suit each patient's unique needs. An integrated approach involving surgery, chemotherapy, radiotherapy, and ongoing research for developing novel treatment modalities can be employed to manage this disease. Given the poor response of pericardial mesothelioma cells to chemotherapy and radiotherapy, surgery remains the primary treatment option. The following indications determine the necessity of surgery: Radical surgery is recommended for localized tumors, while surgery is less effective for late-stage, extensive cancer. Upon diagnosis, our patient exhibited diffuse thick nodular lesions on chest CT and PET/CT imaging, with big tumors located on the anterior and basal regions of the heart. If there is compression of the superior vena cava, palliative surgery is conducted to remove a significant portion of the tumor.

Additionally, a pericardial-pleural drainage window is created to prevent the re-accumulation of fluid, which could lead to cardiac tamponade. This surgical procedure is paired with chemotherapy. Utilizing a treatment protocol consisting of Pemetrexed and Carboplatin following surgery is a justifiable and sensible approach for pleural mesothelioma. The patient's survival duration has been 17 months since the time of diagnosis, indicating a relatively advanced stage of the disease. This outcome is likely attributable, at least in part, to a rational treatment strategy.

Our experiences in this case were key insights in diagnosis and treatment. Key insights gained regarding diagnosing a condition: Diagnosing this condition is challenging, particularly in its early stages, because it is rare and the symptoms are not distinct. Patients are frequently admitted to the hospital due to symptoms associated with pericarditis, pericardial effusion, or symptoms caused by tumor compression and invasion. This condition can be readily mistaken for various disorders such as acute pericarditis, pericardial TB, or cancer spread to the pericardium. Hence, it is imperative to thoroughly assess and synchronize diagnostic techniques, including clinical evaluation, diagnostic imaging such as CT or MRI, PET/CT, along with interventions to extract samples for histopathological examination. Immunohistochemistry should also be employed when necessary for accurate disease diagnosis, in order to avoid overlooking this uncommon condition.

Additionally, other tests should be conducted to eliminate and confirm potential diagnoses before concluding a mesothelioma case. Key insights gained from the course of treatment: The treatment approach for this disease mostly relies on the therapeutic regimen for pleural and peritoneal mesothelioma, as well as insights gained from documented clinical cases that apply to each etiology. In clinical practice, it is essential to effectively integrate surgical procedures, pharmacological interventions, and radiotherapy logically. This should be complemented by symptomatic treatment, physical health assistance, and preventing and managing any potential complications. Special attention should be given to complications associated with cardiac tamponade, heart failure, and arrhythmia. Chemotherapy in these individuals necessitates careful monitoring of cardiovascular function and the prevention of excessive fluid accumulation during treatment.

## 4 Conclusion

This is an exceptionally rare incident. The patient underwent surgical intervention and received chemotherapy as part of their treatment. They have survived for a duration of 17 months, which is almost three times longer than the average survival time observed in other documented cases. Additional reports on cases with pericardial mesothelioma are necessary to have a comprehensive understanding of its nature, enabling early identification and appropriate treatment.

## Consent

Written informed consent was obtained from the patient for publication and any accompanying images. A copy of the written consent is available for review by the Editor-in-Chief of this journal on request.

## Ethical approval

This case report has gotten approval from ethics committee of Military Hospital 103.

## Sources of funding

This research did not receive any specific funding.

## CRediT authorship contribution statement

**Vu Anh Hai:** Conceptualization, Visualization, Writing – original draft. **Nghiem Thi Minh Chau**: Writing – review & editing. **Ho Viet Hoanh:** Writing – review & editing. **Dinh Cong Pho:** Writing – review & editing. **Ha Van Tri:** Resources. **Nguyen Van Nam:** Conceptualization, Visualization, Writing – original draft.

## Guarantor

Assoc Professor, Ph.D. Nguyen Van Nam

Ph.D. Vu Anh Hai

## Research registration number

This study does not require registration.

## Declaration of competing interest

The authors declare no conflicts of interest related to this study.
